# Integrated Metabolomic and Transcriptomic Analysis Reveals the Roles of Cutin, Suberin, and Flavonoid Metabolism in Apple Peel Deterioration Under Non-Bagging Cultivation

**DOI:** 10.3390/plants14213339

**Published:** 2025-10-31

**Authors:** Guiping Wang, Huifeng Li, Ru Chen, Xueping Han, Xiaomin Xue

**Affiliations:** State Key Laboratory of Nutrient Use and Management, Shandong Institute of Pomology, Taian 271000, China; guigui-0530@163.com (G.W.); fenglh79@163.com (H.L.); chenru.8668@163.com (R.C.); hanxuepingrun@163.com (X.H.)

**Keywords:** Fuji apple, non-bagging cultivation, appearance quality, transcriptomics, metabolomics, flavonoid

## Abstract

Non-bagging apple cultivation, which is time-saving, labor-saving, and cost-effective, represents the future direction of apple cultivation in China. However, compared with bagging cultivation, it degrades fruit appearance quality, characterized by rough peels and dull colors, with the underlying physiological and molecular mechanisms remaining unclear. This study used ‘Tianhong 2’ Fuji apples, grafted onto SH dwarfing rootstock, and integrated transcriptomics–metabolomics to explore these mechanisms. Results showed that non-bagging-cultivated apple peels had higher chlorophyll and carotenoid contents but lower anthocyanin content than those of bagging-cultivated ones. Transcriptome sequencing identified 1571 differentially expressed genes (DEGs: 1269 upregulated, 302 downregulated). Functional analysis revealed that the decline in fruit appearance quality was primarily associated with secondary metabolite biosynthesis, lipid metabolism, and carbohydrate metabolism, and 34 candidate genes were identified. Metabolomic analysis detected 394 differentially expressed metabolites (DEMs), 38 of which were closely related to the non-bagging-induced appearance degradation, mainly lipids, organic oxygen compounds, and organic acids and their derivatives. Integrated analysis of DEGs and DEMs indicated the involvement of multiple critical metabolic pathways, including cutin, suberin and wax biosynthesis; starch and sucrose metabolism; cyanoamino acid metabolism; and phenylpropanoid and flavonoid biosynthesis. Compared with bagging-cultivated apples, non-bagging-cultivated apples exhibited faster starch degradation and higher soluble sugar accumulation. Additionally, the accumulation of specific metabolites [e.g., quercetin (HMDB0005794, HMDB03249, LMPK12112097), and suberin components (LMFA01170020, LMFA01050437, HMDB0031885)], along with elevated organic acid levels, contributed to peel roughness and dull coloration. These findings further enrich the theoretical basis for the formation of fruit quality in Fuji apples under non-bagging cultivation and provide valuable theoretical guidance for the practical application of this cultivation mode.

## 1. Introduction

Apple (*Malus domestica* Borkh.) is one of the most widely cultivated and highest-yielding fruit tree species globally. China, as the world’s largest apple producer and consumer, ranks first in both production and cultivation area worldwide [1]. Apple production plays a crucial role in enhancing agricultural efficiency, increasing farmers’ income, advancing China’s agricultural supply-side structural reform, facilitating the rural revitalization strategy, and promoting targeted poverty reduction [2,3]. Fuji apple, renowned for its large fruit size, bright color, and excellent storability, is the most widely cultivated apple variety in China, accounting for over 70% of the country’s total apple cultivation area and total production. Therefore, the Fuji apple industry holds a dominant position in the Chinese apple industry [4,5]. Since its introduction, the Fuji apple has generally been cultivated with bagging to control fruit borers and rust [4,6]. The promotion and application of bagging technology have historically played a vital role in improving the market competitiveness of Chinese apples and increasing farmers’ income.

In recent years, with the intensification of population aging and the transfer of rural labor to urban areas, labor shortages and intense labor competition have become prevalent during the bagging season, keeping farmers’ production costs high [7]. Furthermore, issues such as reduced internal fruit quality and aggravated physiological diseases associated with bagging cultivation [8,9] have driven growing calls for the adoption of non-bagging cultivation of Fuji apples. Research on non-bagging cultivation has emerged as a research hotspot in the fields of apple breeding, cultivation, and plant protection, and represents the future development direction of China’s apple industry. In 2020, the apple non-bagging cultivation technology was listed as one of the top ten leading technologies by China’s Ministry of Agriculture [10,11]. Extensive research and trials on apple non-bagging cultivation have been conducted in China. Recent research on bagged versus unbagged cultivation has primarily advanced along two tracks. The first focuses on agronomic and pest management outcomes. Studies have confirmed that unbagged cultivation significantly reduces labor costs and alleviates issues such as internal quality degradation and physiological disorders like bitter pit that are often associated with bagging [8,9]. Consequently, research has intensified on breeding new apple varieties suitable for unbagged conditions [12,13,14] and developing integrated strategies for pest and disease control [15,16,17]. The second, more recent track employs physiological and molecular approaches to understand the underlying mechanisms. Efforts have been made to improve fruit appearance in unbagged systems using techniques like biofilm agents [18,19,20], and studies have started to link pre-harvest bagging treatments with specific changes in fruit quality parameters [20]. However, a critical knowledge gap remains: a comprehensive understanding of the molecular and metabolic mechanisms directly responsible for the deterioration of peel quality (characterized by roughness and dull coloration) in unbagged ‘Fuji’ apples is still lacking.

Notably, limited research has been reported on the molecular mechanisms underlying the unsatisfactory appearance quality of non-bagging-cultivated apples (e.g., rough peels, dull coloration, and reduced smoothness). Therefore, this study aimed to uncover the causes and potential mechanisms behind these issues. Using the ‘Tianhong 2’ Fuji apple cultivar as the experimental material, we performed an integrated analysis of physiological, biochemical, transcriptome, and metabolome data to reveal the mechanism underlying the decline in appearance quality of Fuji apples under non-bagging cultivation, thereby providing theoretical guidance and technical support for promoting the application of non-bagging cultivation.

## 2. Results

### 2.1. Effects of Non-Bagging and Bagging Cultivation on the Appearance Quality of Fuji Apples

**Figure 1:**
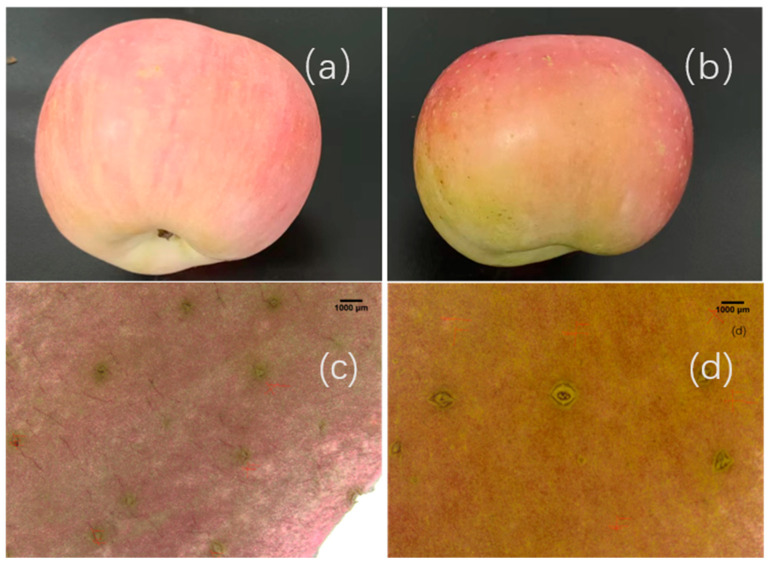
Effects of non-bagging and bagging on appearance characteristics of the Fuji fruit. (**a**) Representative appearance of a bagging-cultivated apple; (**b**) Representative appearance of a non-bagging-cultivated apple; (**c**) Micrograph of fruit dots on the peel of a bagging-cultivated apple. (**d**) Micrograph of fruit dots on the peel of a non-bagging-cultivated apple. The red font in the (**c**,**d**) represents the length and width data of the fruit dots (μm).

As shown in Figure 1, non-bagging-cultivated Fuji apples had a slightly flattened shape, heavier coloration, and obvious yellowish-green deposits on the background color. In contrast, bagging-cultivated apples exhibited bright and ruddy coloration with a pale-yellow background color (Figure 1a,b).

Non-bagging-cultivated apples had larger fruit dots with lower density and darker, more noticeable color. Specifically, the density, length, and width of the fruit dots in non-bagging-cultivated apples were 4.71 per cm^2^, 881.95 μm, and 696.04 μm, respectively (Table 1). For bagging-cultivated apples, these parameters were 5.15 per cm^2^, 640.14 μm, and 446.17 μm, respectively. Non-bagging cultivation significantly reduced the appearance quality of Fuji apples, as evidenced by a significant decrease in both the coloration index and the smoothness index. The coloration index and smoothness index of non-bagging-cultivated apples were 79.55% and 70.45%, respectively, while those of bagging-cultivated apples were 90.91% and 86.36%, respectively (Table 2).

Further measurements of the peel color difference between non-bagging and bagging-cultivated Fuji apples were conducted, and the results were shown in Table 2. Compared with bagging-cultivated apples, non-bagging-cultivated Fuji apples exhibited significantly lower lightness (L*), color intensity (C), and comprehensive color difference (h°), and the a* value (representing red color) also was significantly lower.

### 2.2. Effects of Non-Bagging and Bagging Cultivation on the Ultrastructure of Fuji Apple Peel

**Figure 2:**
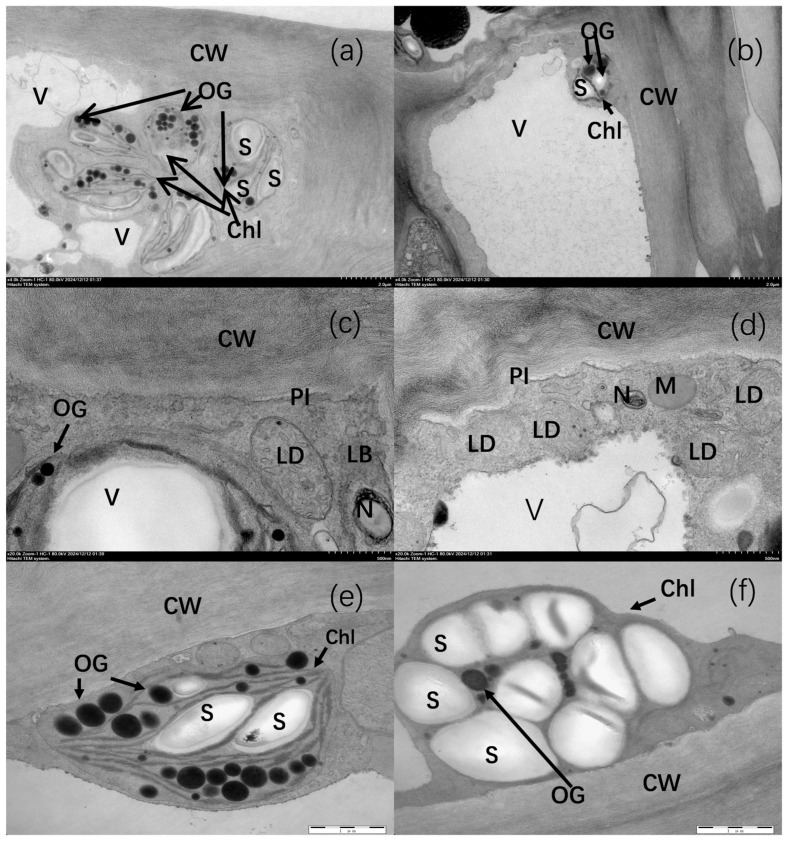
Effects of non-bagging and bagging on the ultrastructure of Fuji apple peel ((**a**,**b**): ×4000 magnification, (**c**,**d**): ×20,000 magnification, (**e**,**f**): ×30,000 magnification; (**a**,**c**,**e**): non-bagging; (**b**,**d**,**f**): bagging), CW—cell wall, Chl—chloroplast, S starch grain, OG—osmiophilic granule, V—vacuole, LD—lipid droplet, N—cell nuclei, PI—plasmolysis. Arrow: the major change in lamella.

As shown in Figure 2, the cell walls (CW) of pericarp cells in non-bagging-cultivated apples were irregular, with significant light and dark variations, and the cell walls were thickened. In contrast, the cell walls of pericarp cells in bagging-cultivated apples were relatively smooth, with neatly arranged fibers and gentle light-dark transitions (Figure 2a,b). Additionally, pericarp cells in non-bagging-cultivated apples contained abundant osmiophilic granules (OG), which were scattered in the chloroplast stroma. In comparison, bagging-cultivated apples had relatively fewer osmiophilic granules, which were distributed in the interstices of starch grains (Figure 2a–d).

Furthermore, pericarp cells of non-bagging-cultivated apples contained a large number of chloroplasts (Figure 2a). These chloroplasts (Chl) had relatively intact structures, with clearly visible thylakoid lamellae, exhibiting the typical internal membrane characteristics of chloroplasts (Figure 2c). In contrast, chloroplast structures in bagging-cultivated apples were compressed by a large number of starch grains (S), resulting in indistinct thylakoid lamellae. Moreover, the shape of the chloroplasts appeared irregular due to starch grain accumulation (Figure 2d).

In pericarp cells of non-bagging-cultivated apples, there were fewer lipid droplets (LD), and mitochondria (M) were small in size and low in quantity (Figure 2e). On the other hand, bagging-cultivated apples had relatively abundant LD, and mitochondria were more numerous and distributed more dispersedly (Figure 2f). Vacuoles (V) in non-bagging-cultivated apples had relatively regular boundaries (Figure 2e), whereas vacuole boundaries in bagging-cultivated apples showed irregular invaginations or protrusions (Figure 2f). Notably, cell walls in bagging-cultivated apples exhibited obvious plasmolysis, while this phenomenon was significantly less pronounced in non-bagging-cultivated apples (Figure 2e,f).

### 2.3. Effects of Non-Bagging and Bagging Cultivation on the Pigment Content of Fuji Apple Peel

#### 2.3.1. Chlorophyll and Carotenoid Content in Fruit Peel

As shown in Table 3, the chlorophyll and carotenoid contents in the peels of non-bagging-cultivated Fuji apples were significantly higher than those in bagging-cultivated apples. Specifically, the chlorophyll and carotenoid contents in the pericarp of non-bagging-cultivated Fuji apples were 0.019 mg/g FW and 0.016 mg/g FW, respectively, while those in bagging-cultivated apples were 0.002 mg/g FW and 0.008 mg/g FW, respectively.

#### 2.3.2. Total Anthocyanin and Total Flavonoid Content in Fuji Apple Fruit Peel

**Figure 3:**
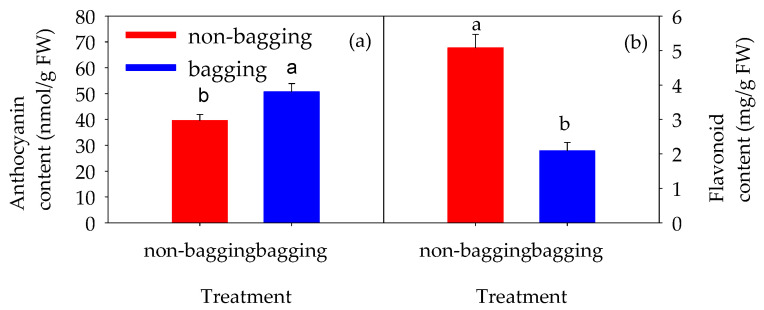
Effects of non-bagging and bagging cultivation on anthocyanin content (**a**) and flavonoid content (**b**) in Fuji apple fruits peel. Data are presented as the mean ± SE (n = 3). Error bars represent S.E. of three replicates. Different lowercase letters indicate significant differences between treatments as determined by Duncan’s test (*p* < 0.05).

Further measurements of anthocyanins and total flavonoids in the fruit peels were conducted. As shown in Figure 3, the anthocyanin content in non-bagging-cultivated apples was significantly lower than that in bagging-cultivated apples (*p* < 0.05); in contrast, the flavonoid content in the peels of non-bagging-cultivated Fuji apples was significantly higher than that in bagging-cultivated apples (*p* < 0.05).

### 2.4. Transcriptomic Analysis of Non-Bagging and Bagging-Cultivated Fuji Apple Peels

#### 2.4.1. Transcriptome Sequencing and Quality Control of Fuji Apple Peel Samples

To investigate the differences in appearance quality between non-bagging and bagging-cultivated apples, transcriptome sequencing was performed on mature apple peels, and the results are shown in Table 4. After filtering out low-quality data, a total of 39.54 Gb of clean data was obtained. The effective data size of each sample ranged from 6.08 to 6.92 Gb, and the Q30 base ratio ranged from 93.80 to 95.00%. The average GC content was 48.73% (Table 4), indicating high-quality transcriptome data.

**Figure 4:**
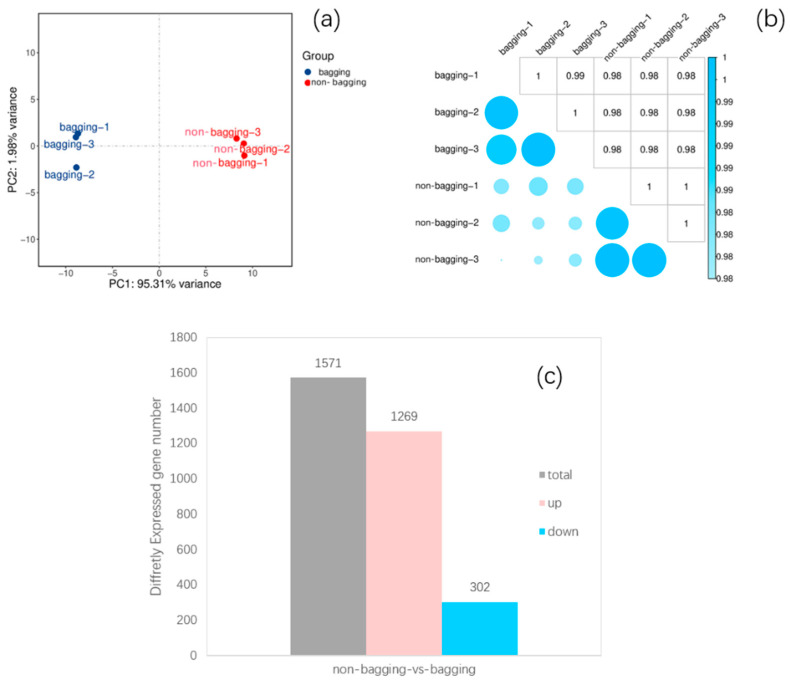
Transcriptomic profiling of non-bagging and bagging-cultivated Fuji apple peels. (**a**) Principal component analysis (PCA) showing clear separation between the two cultivation methods. (**b**) Correlation heatmap of biological replicates. (**c**) Volcano plot of differentially expressed genes (DEGs) between non-bagging and bagging groups.

As shown in Figure 4, a total of 35,590 genes were annotated (Counts: Anno) in the entire project. To identify key factors in the transcriptome data, principal component analysis (PCA) was used to visualize the differences between non-bagging and bagging treatments (Figure 4). PCA results showed that the first two principal components (PC1, PC2) can clearly distinguish between the two treatments: samples from the same treatment were clustered together, while samples from different treatments were distinctly separated. Additionally, the correlation heatmap (Figure 4b) showed a high correlation (correlation coefficient > 0.98) between biological replicates in the same group, indicating good sample reproducibility. The first and second principal components explained 97.29% of the total variance (Figure 4a), confirming the rationality of sample selection and the high reliability of the experiment. A volcano plot of 1571 DEGs (Figure 4c) showed that, compared with bagging-cultivated apples, non-bagging-cultivated apples had 1269 upregulated and 302 downregulated unigenes.

#### 2.4.2. Identification of DEGs Between Non-Bagging and Bagging Fuji Apple Peels

**Figure 5:**
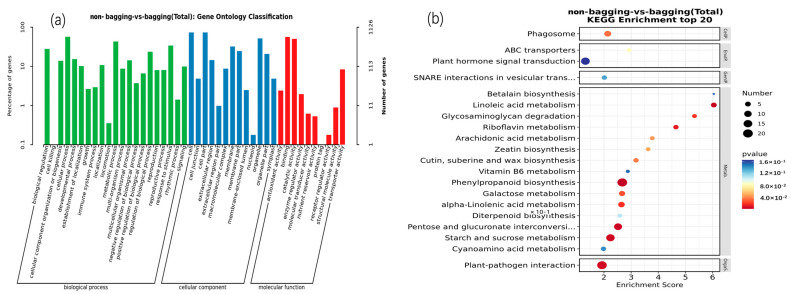
Enrichment analysis of DEGs based on Gene Ontology (GO) and Kyoto Encyclopedia of Genes and Genomes (KEGG). (**a**) GO enrichment analysis, (**b**) KEGG pathway analysis.

GO functional enrichment analysis was performed on the DEGs. As shown in Figure 5a, the DEGs were enriched in three major categories: Biological Process (BP), Cellular Component (CC), and Molecular Function (MF). Among these, the CC category contained the largest number of enriched DEGs, most of which were associated with cell, cell part, organelle, and membrane-related functions. In the BP category, DEGs were mainly enriched in biological processes such as cellular processes, metabolic processes, response to stimuli, and regulation of biological process; in the MF category, DEGs were primarily enriched in binding activity and catalytic activity. KEGG pathway enrichment analysis was conducted on the DEGs (Figure 5b). Results showed that most DEGs between the non-bagging and bagging treatments were enriched in the following pathways: phenylpropanoid biosynthesis, linoleic acid metabolism, pentose and glucuronate interconversions, starch and sucrose metabolism, and plant-pathogen interaction.

#### 2.4.3. Screening of Candidate Genes Related to Appearance Quality Deterioration and qRT-PCR Validation

Based on GO functional enrichment and KEGG pathway enrichment analysis of DEGs, it can be concluded that the rough peel and dull coloration of non-bagging-cultivated Fuji apples may be mainly associated with metabolic processes such as secondary metabolite biosynthesis, lipid metabolism, carbohydrate metabolism, and environmental adaptation. Candidate genes were thus screened from these functional categories, and the results are shown in Table 5.

The darkening of apple peel color is associated with differential accumulation of secondary metabolites in the peel: CYP75B2 (MD14G1210700), a key enzyme in flavonoid biosynthesis, catalyzes the 3′-hydroxylation of the B-ring in flavonoids, promoting the conversion of kaempferol to quercetin. This process affects flavonoid structural diversity, regulates flower color and stress resistance, and the upregulation of CYP75B2 increases flavonol production. The upregulation of LAR (MD13G1046900) enhances the synthesis of leucoanthocyanidin reductase, which facilitates the conversion of leucoanthocyanidins to catechins and alters flavonoid metabolic flux. The upregulation of both genes promotes flavonol biosynthesis, which deepens peel color and enhances stress resistance; their high expression is consistent with the dark peel color of non-bagging-cultivated apples. CCD4 (MD13G1090300) is responsible for catalyzing the cleavage of carotenoids (e.g., β-carotene), and its downregulation may reduce carotenoid degradation. NCED1 (MD10G1194200), a rate-limiting enzyme in abscisic acid (ABA) biosynthesis, promotes ABA accumulation when upregulated. ABA may inhibit the expression of carotenoid-degrading genes (e.g., CCD4), further increasing carotenoid content. Together, these two genes contribute to peel color darkening (e.g., from green to yellow). The downregulation of CYP84A1-like (MD02G1136000) reduces ferulic acid production, potentially decreasing the biosynthesis of guaiacyl (G)-type lignin (with ferulic acid as a precursor). Lignin is a critical component of the peel cell wall, directly affecting peel firmness, structural integrity, and storage performance (e.g., resistance to fruit cracking and mechanical damage). The upregulation of COMT increases sinapic acid production, which may enhance the biosynthesis of syringyl (S)-type lignin, with sinapic acid as its precursor, thereby affecting peel firmness and structure.

The dull coloration of apple peel involves redox processes that counteract excessive reactive oxygen species (ROS) generation. For instance, peroxidases (MD01G1162700, MD15G1252300) are associated with hydrogen peroxide-related oxidation reactions, catalyzing the oxidation of various substrates (e.g., phenols, amines). The upregulation of these genes in the peel catalyzes the oxidation of phenolics to quinones, which further polymerize to form dark-colored substances such as melanin. The downregulation of polyphenol oxidase (PPO, MD05G1320800) is speculated to be related to the negative regulation of quinones. The downregulation of amine oxidase 4 (CAO1, MD14G1172500)—which catalyzes the oxidative decomposition of polyamines such as putrescine and spermidine-leads to polyamine accumulation, enhancing antioxidant defense (e.g., free radical scavenging) and regulating stress responses.

Genes related to lipid metabolism: LOX2.1 (MD11G1023100), which is strictly localized in chloroplasts, is directly involved in chloroplast membrane lipid metabolism and the production of jasmonic acid (JA) precursors. When apple peels are exposed to ultraviolet (UV) radiation, LOX2.1 may be preferentially activated, initiating antioxidant defense via the JA signaling pathway. The upregulation of LOX6 (MD16G1113200) increases cell membrane permeability. These changes are consistent with the high degree of cell wall degradation observed in non-bagging-cultivated apple peels (Figure 2a). Additionally, cytochrome P450 94A1 (MD10G1190400), brassinosteroid-related acyltransferase 1-like (BAT1, MD08G1058900), and probable peroxygenase 4 (PXG4, MD13G1029500) are involved in fatty acid metabolism and contribute to the formation and defensive functions of cutin and suberin.

Carbon metabolism is enhanced in the peels of non-bagging-cultivated apples: the downregulation of ribulose bisphosphate carboxylase small chain (MD02G1011600) and ribulose-1,5-bisphosphate carboxylase/oxygenase large subunit (MD13G1247200) reduces the photosynthetic capacity of peel cells, thereby, decreasing CO_2_ fixation and glucose synthesis. Carbon metabolism thus shifts from “photosynthetic production” to “storage or catabolic utilization”, which is consistent with the phenotypic change in the peel from green (chlorophyll-containing) to red or yellow (dominated by carotenoids/anthocyanins). ADG2 (MD13G1186100)—a rate-limiting enzyme in starch synthesis—and BAM3 (MD04G1056200), related to starch degradation, may be involved in the conversion of starch to soluble sugars during apple ripening (e.g., starch disappearance in ‘Orin’ apples in the late ripening stage). The upregulation of SPS4 (MD05G1006400, probable sucrose-phosphate synthase 4) primarily reflects enhanced sucrose synthesis capacity in fruits, which is closely associated with carbon metabolism regulation, quality formation, and environmental adaptation in fruits. The upregulation of At4g24780 (MD14G1167100), PME53 (MD02G1067000), and At1g64390 (MD14G1128000) indicates the coordinated degradation of pectin and cellulose components in the cell wall, which disrupts the “skeletal” structure of the cell wall. CHIT (MD02G1120300), which degrades chitin, may be involved in cell wall softening (promoting fruit ripening) or defense against fungal pathogens (e.g., apple anthracnose).

The upregulation of environmental adaptation-related genes CNGC1 (MD07G1292500), CML41 (MD08G1037100), EIX2 (MD02G1271900), and CML27 (MD17G1257900) primarily reflects that the fruits may be in a stress-responsive state, with cell signal transduction (especially the calcium signaling pathway) and defense mechanisms activated.

Changes in energy metabolism genes indicate a transition of the peel from a photosynthetic organ to a protective or storage organ. In the peel of non-bagging-cultivated apples, NADP-dependent malic enzyme isoform X2 (MD15G1091000) is upregulated, which may compensate for the reduced photosynthetic capacity.

ABC transporters are a class of transmembrane proteins located on cell membranes. The ABCB family is often involved in the transport of auxins and phytoalexins (such as flavonoids and terpenoids), which influence fruit peel color. The upregulation of ABC transporter B family member 15 (MD17G1042800) and ABC transporter B family member 9 (MD10G1268400) may be involved in the regulation of these substances.

**Figure 6:**
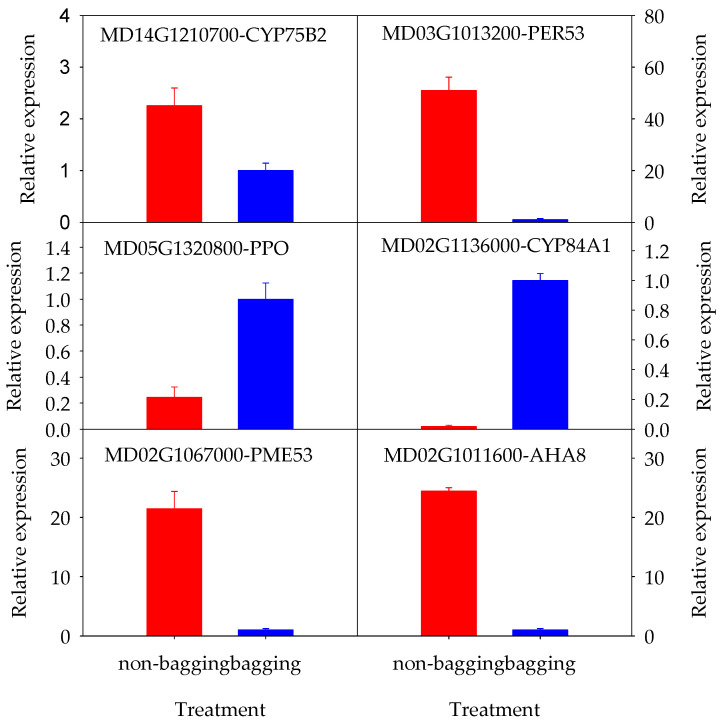
Validation of RNA-seq data by qRT-PCR. Error bars represent the SE (n = 3).

Six DEGs (MD05G1320800, MD14G1210700, MD02G1136000, MD02G1067000, MD02G1011600, MD03G1013200) were randomly selected from the screened candidate genes for qRT-PCR detection and analysis. The results are shown in Figure 6. The expression patterns of these six candidate genes were consistent with the RNA-seq results, confirming the reliability of the transcriptome data.

### 2.5. Metabolome Analysis of Non-Bagging and Bagging Fuji Apple Peels

**Figure 7:**
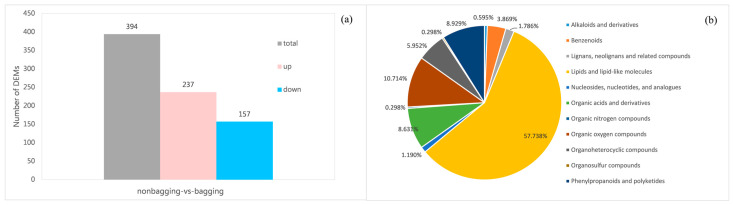
Statistical diagram of differentially expressed metabolites (DEMs) (**a**) and DEMs classification (**b**).

A total of 7246 metabolites were identified (Supplemental Table S1), among which 838 DEMs were detected in the non-bagging vs. bagging comparison (VIP > 1, *p*-value < 0.05). Further screening of DEMs was performed using the criteria of VIP > 1.0 and fold change (FC) > 2 or FC < 0.5. Ultimately, 394 DEMs were identified between non-bagging and bagging groups, including 237 upregulated metabolites and 157 downregulated metabolites (Figure 7a). The screened DEMs were compared and analyzed against the Human Metabolome Database (HMDB) to obtain their classification information, with the results presented in Figure 7b. Among the 394 DEMs, 58 were unclassified, while the majority of the remaining metabolites belonged to “lipids and lipid-like molecules”, accounting for 57.74%. The other major categories, in descending order of proportion, were “Organic oxygen compounds” (10.71%), “Phenylpropanoids and polyketides” (8.93%), and “Organic acids and derivatives” (8.63%). These findings indicate that the metabolites altered in Fuji apple peels between non-bagging and bagging cultivation are mainly lipids and lipid-like molecules, organic oxygen compounds, phenylpropanoids and polyketides, as well as organic acids and derivatives.

**Figure 8:**
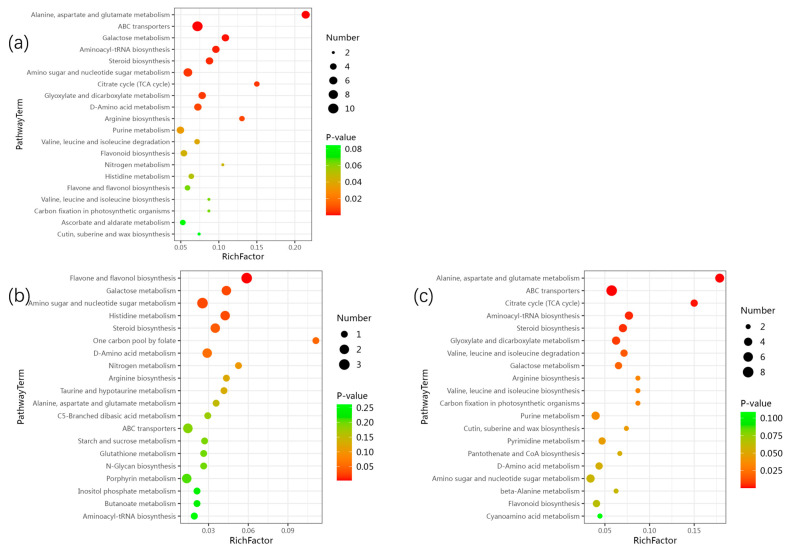
KEGG enrichment analysis of DEMs. (**a**) Top 20 enriched pathways for all DEMs, (**b**) Top 20 pathways for upregulated DEMs and (**c**) Top 20 pathways for downregulated DEMs (non-bagging vs. bagging).

The screened DEMs (Table S2) were mapped to the KEGG pathway database. Based on the mapping results, information regarding the metabolic pathways involved in these DEMs was obtained. The identified metabolic pathways included alanine, aspartate and glutamate metabolism, ABC transporters, galactose metabolism, aminoacyl-tRNA biosynthesis, and steroid biosynthesis. These findings indicate that, compared with bagging cultivation, the DEMs in the peel of non-bagging-cultivated apples are mainly involved in the aforementioned metabolic pathways (Figure 8a). The main pathways for significant enrichment of upregulated metabolites include flavone and flavonol biosynthesis, galactose metabolism, amino sugar and nucleotide sugar metabolism, histidine metabolism, and steroid biosynthesis (Figure 8b); the main pathways for significant enrichment of downregulated metabolites include alanine, aspartate and glutamate metabolism, ABC transporters, citrate cycle (TCA cycle), aminoacyl-tRNA biosynthesis, steroid biosynthesis, glyoxylate and dicarboxylate metabolism (Figure 8c).

Based on HMDB compound classification and KEGG functional pathway analysis of DEMs, it was found that the metabolites associated with the rough peel and dull coloration of non-bagging-cultivated Fuji apples are mainly membrane lipids, organic acids, organic oxygen compounds, phenylpropanoids and polyketides, and heterocyclic compounds. A total of 38 DEMs potentially related to rough peel and dull coloration were screened out, among which membrane lipids, organic acids, organic oxygen compounds, phenylpropanoids and polyketides, and heterocyclic compounds accounted for 12, 11, 1, 10, and 11 respectively; in addition, there were 3 nucleoside-related metabolites (Table 6).

Non-bagging-cultivated fruits are more susceptible to environmental stress, and changes in lipid metabolites are among the early manifestations of environmental stress response.

δ8,14-Sterol (HMDB0006928) is associated with apple resistance to environmental stresses such as diseases and pests; its upregulation helps enhance cell membrane stability. Lactosamine (HMDB0006591) increases cell wall thickness. Asparagoside B (HMDB0029315) facilitates resistance to adverse external factors. Meanwhile, flavonol glycosides are involved in the formation of yellow or green color in apple peels, and their accumulation may act synergistically with chlorophyll degradation or carotenoid synthesis. Quercetin derivatives (LMPK12112185, LMPK12112087, and LMPK12112155) belong to flavonoids and possess antioxidant and antibacterial activities; their upregulation is likely a defensive response of apple peels to environmental stresses such as ultraviolet radiation and pathogen invasion. Changes in S-(3-Methylbutanoyl)-dihydrolipoamide-E (HMDB0006867) are involved in intracellular energy metabolism and substance transformation processes. Changes in cholesteryl α-D-glucoside (LMST01010173) may affect the fluidity and permeability of cell membranes, thereby influencing the transport of substances between the inside and outside of cells as well as signal transduction; the upregulation of both these metabolites is conducive to substance transport and transformation.

The contents of organic acid metabolites, such as L-Glutamic acid (HMDB0000148) and L-Aspartic acid (HMDB00191, HMDB0006483), are downregulated. Most of these metabolites are associated with nitrogen metabolism. Their downregulation may indicate that the non-bagging cultivation environment impairs the absorption, transport, and utilization of nitrogen in fruits. This further hinders the synthesis of macromolecules like proteins and nucleic acids, leading to irregular cell arrangement. Such cellular disorder is manifested on the fruit surface as a loss of smooth texture, resulting in a rough appearance.

D-Galactose (HMDB0000143) is a component of cell wall polysaccharides such as pectin. Its downregulation may lead to softer fruit texture, which is consistent with the lower pectin content in the peels of non-bagging-cultivated fruits (non-bagging: 22.13 ± 0.91 g/kg FW; bagging: 25.51 ± 1.32 g/kg FW). Reduced pectin content decreases cell-to-cell adhesion, rendering the peel tissue more susceptible to water loss. This subsequent water loss further contributes to the dull coloration of the peel.

Rutin (HMDB03249) and quercetin (HMDB0005794) both belong to flavonoids. Rutin and quercetin themselves exhibit a characteristic color, typically light yellow or yellow. Their accumulation in the peel can directly impart a yellow hue to the peel, leading to varying degrees of yellow coloration. Quercetin 3-neohesperidoside (LMPK12112097), a glycoside form of quercetin, also falls into the flavonoid category and is usually light yellow; it can directly contribute a yellow tone to the peel. The upregulation of these three metabolites enhances the yellow hue of the peel, which may be one of the reasons for the darker coloration of the peel in non-bagging cultivation. The downregulation of naringin dihydrochalcone (HMDB0255461), catechin 5-glucoside (HMDB0037948), and cinnamic acid (HMDB0000567) may lighten the color of apple peels. This downregulation weakens the promotional effect on the synthesis of pigments such as anthocyanins, reducing the expression of bright colors (e.g., red) and thus exerting a negative impact on apple peel coloration. Dehydroascorbic acid (HMDB0001264) is the oxidized form of ascorbic acid (vitamin C). Its downregulation may shift the redox balance in peel cells toward an oxidized state, which is unfavorable for anthocyanin formation—this is also one of the factors contributing to the dull appearance of the peel.

Deoxycytidine monophosphate (dCMP, HMDB0001202) and 2′-Deoxyguanosine 5′-monophosphate (HMDB0001044) are raw materials for DNA synthesis. 3′-Adenosine monophosphate (3′-AMP, HMDB0003540) is a nucleotide that plays a role in intracellular energy metabolism and signal transduction. The downregulation of the first two (dCMP and 2′-Deoxyguanosine 5′-monophosphate) as well as 3′-AMP may imply that DNA synthesis in apple peel cells is slowed down, and cell division activity may be weakened. This suggests that more energy and substances are diverted to metabolic processes related to fruit growth and ripening. Uridine diphosphate-D-galactose (UDP-D-galactose, HMDB00302) is an important sugar nucleotide involved in polysaccharide synthesis. Its upregulation indicates enhanced polysaccharide synthesis and metabolism in apple peels, which contributes to strengthening the structure of cell walls.

### 2.6. Joint Analysis of DEGs and DEMs

Based on the KEGG pathway annotation information of DEGs and DEMs, the DEGs and DEMs involved in the same KEGG pathway were correlated to clarify the relationship between DEGs and DEMs related to the peels of non-bagging-cultivated Fuji apples (with lower appearance quality than those of bagging-cultivated apples).

#### 2.6.1. Starch and Sucrose Metabolism

**Figure 9:**
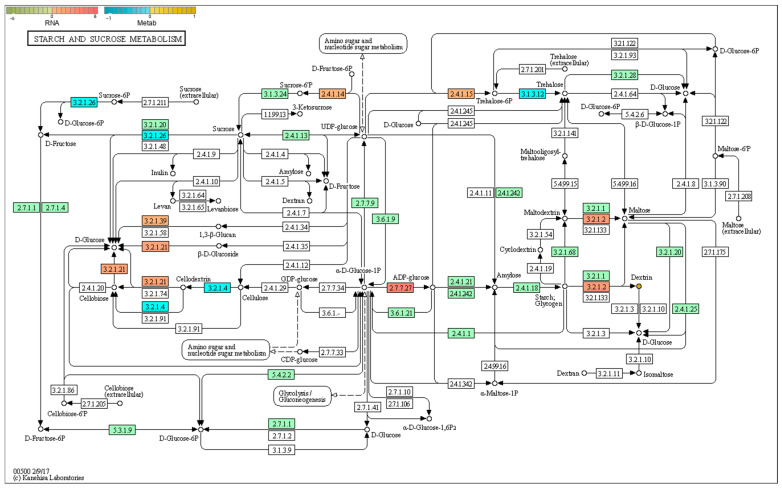
Joint pathway analysis of DEGs and DEMs in non-bagging vs. bagging Fuji apple peels: Starch and sucrose metabolism pathway. The gene expression levels of differentially expressed genes (DEGs) are represented by rectangles, and adjacent heatmaps (red to green scales) show the expression values of the samples. Differentially expressed metabolites (DEMs) are represented by solid circles, with yellow indicating upregulation and blue indicating downregulation. The color scale represents the level of expression (log_2_ (FoldChange)).

As shown in Figure 9, genes related to the starch-sucrose metabolism pathway—such as glucose-1-phosphate adenylate transferase (MD13G1186100) and amylase (MD04G1056200)—were regulated, leading to the upregulation of dextrin content. The upregulation of dextrin indicates accelerated starch degradation, which is consistent with the observation of relatively fewer starch granules in the peels of non-bagging-cultivated fruits (Figure 2). Meanwhile, an increase in dextrin content may provide abundant raw materials and energy for the synthesis of cell wall substances or flavonoids, which is beneficial for the accumulation of total flavonoids (Figure 3).

#### 2.6.2. Flavonoid Biosynthesis

**Figure 10:**
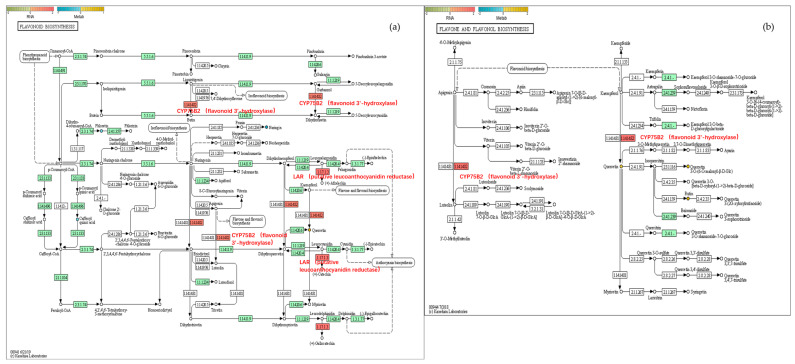
Joint pathway analysis of DEGs and DEMs in non-bagging vs. bagging Fuji apple peels: (**a**) Flavonoid biosynthesis pathway, (**b**) Flavone and flavonol biosynthesis pathway. The gene expression levels of differentially expressed genes (DEGs) are represented by rectangles, and adjacent heatmaps (red to green scales) show the expression values of the samples. Differentially expressed metabolites (DEMs) are represented by solid circles, with yellow indicating upregulation and blue indicating downregulation. The color scale represents the level of expression (log2 (FoldChange)). The key enzyme steps have now been fully labeled (e.g., 1.141482: CYP75B2 (flavonoid 3′-hydroxylase)), directly linking DEGs to metabolic pathways.

As shown in Figure 10, key genes involved in flavonoid metabolism, such as flavonoid 3′-hydroxylase CYP75B2 (MD14G1210700) and putative leucocyanidin reductase (MD13G1046900), were upregulated, leading to an increase in the flavonoid compound quercetin (Figure 10a). The contents of other flavonoid compounds such as rutin and quercetin 3-O-malonylglucoside also increased significantly (Figure 10b). These substances themselves have a characteristic color (usually yellow), and their accumulation affects the background color of the fruit peel, resulting in different shades from light yellow to light brown.

#### 2.6.3. Cutin, Suberin and Wax Biosynthesis

**Figure 11:**
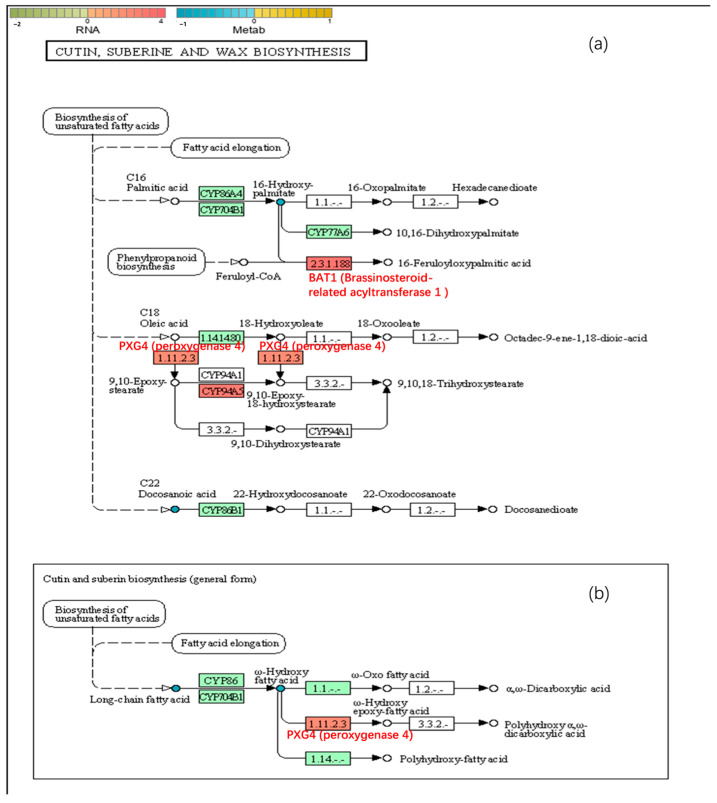
Joint pathway analysis of DEGs and DEMs in non-bagging vs. bagging Fuji apple peels: (**a**) Cutin, suberin and wax biosynthesis pathway, (**b**) cutin and suberin biosynthesis pathway. The gene expression levels of differentially expressed genes (DEGs) are represented by rectangles, and adjacent heatmaps (red to green scales) show the expression values of the samples. Differentially expressed metabolites (DEMs) are represented by solid circles, with yellow indicating upregulation and blue indicating downregulation. The color scale represents the level of expression (log_2_ (Fold Change)). The key enzyme steps have now been fully labeled (e.g., 2.3.1.188: BAT1 (brassinosteroid related acyltransferase 1)), directly linking DEGs to metabolic pathways.

As shown in Figure 11, compared with bagging cultivation, the key genes in the fatty acid synthesis pathway—CYP94A1 (MD10G1190400), PXG4 (MD13G1029500), and BAT1 (MD08G1058900)—were upregulated in non-bagging-cultivation, resulting in a decrease in the contents of metabolites 16-hydroxyhexadecanoic acid (HMDB0006294) and behenic acid. The fatty acid metabolism pathway is the basis for the synthesis of cutin, suberin, and wax, providing energy and carbon sources for the synthesis of cutin and suberin; it can also be transformed into specialized fatty acids suitable for cuticular suberin synthesis through steps such as elongation and modification in the fatty acid synthesis pathway. This corresponds to an increase in the content of suberin components (Table 6). Cuticular suberin plays an important role in plants’ resistance to external biotic and abiotic stresses (e.g., pathogen invasion and water loss). This is consistent with the fact that non-bagging-cultivated fruits are more susceptible to environmental stresses (including abiotic stress)—non-bagging-cultivation promotes the metabolism of related fatty acids and the synthesis of cuticular suberin, the barrier function of the fruit peel is enhanced. This is also one of the reasons for the rough peel and dull coloration.

## 3. Discussion

### 3.1. The Appearance Quality of Fuji Apples Under Non-Bagging Cultivation Is Significantly Lower than That Under Bagging Cultivation

Compared with bagging cultivation, non-bagging cultivation reduces the appearance quality of Fuji apples, manifested as rough peels and dull coloration (Figure 1). This is the result of a series of changes in peel ultrastructure (Figure 2) and peel-related substances (Table 3 and Figure 3).

In contrast to bagging cultivation, the fruit dots on the peels of non-bagging-cultivated Fuji apples are large and uneven (Figure 2), which is consistent with the lower smoothness index (Table 2). This finding aligns with the research results of previous studies [21,22] and also coincides with the research on pears [23]. It is speculated that since non-bagging-cultivated fruits are directly exposed to the variable external environment and affected by diseases and insect pests, their metabolism and cell division are impacted, which in turn affects the size and uniformity of fruit dots. Furthermore, it should be noted that the specific rootstock system used in this study (SH/Malus robusta) might have also contributed to the observed peel characteristics, as rootstocks are known to modulate the tree’s overall vigor and resource allocation, potentially influencing fruit skin development. Future studies could specifically investigate the interaction between cultivation practices and different rootstock genotypes.

Compared with bagging cultivation, the coloration index and a* value (representing red in color difference measurements) of non-bagging-cultivated Fuji apples are significantly reduced (Table 2). Peel color is formed by the interaction of pigments such as anthocyanins, chlorophyll, carotenoids, and flavonoids; different contents of these pigments in the peel result in different “hues” and “tones” [5,24]. The results of this study show that the contents of chlorophyll and carotenoids in the peel of non-bagging-cultivated Fuji apples are significantly higher than those in bagging-cultivated apples (*p* < 0.05) (Table 3), while the anthocyanin content is significantly lower (*p* < 0.05) (Figure 3a). This is consistent with the findings of previous researchers [25,26]. It is inferred that the different ratios of pigment contents in the peel are one of the reasons for the reduced appearance quality of non-bagging-cultivated Fuji apples. To clarify the specific molecular mechanism behind this, peel transcriptome sequencing and metabolome analysis were further conducted.

### 3.2. The Reduction in Appearance Quality Is Related to Differences in the Expression of Genes Involved in the Synthesis of Peel Resistance-Related Substances

The results of this study demonstrated that the deterioration in appearance quality of non-bagging-cultivated Fuji apples—such as uneven fruit dots on the peel and decreased smoothness—is significantly associated with the differential expression of genes related to peel resistance substance synthesis [27] (Figure 5).

Suberin-related substances in fruit peel are complex high-molecular-weight polymers. When plants are attacked by pathogens or injured, suberin accumulates substantially in the cell walls of periderm cells, forming a hydrophobic barrier. When suberin components accumulate in large quantities in the peel, the number of suberized cells increases. The cell walls of these cells are highly suberized and stack in multiple layers to form a thick suberin layer, which in turn makes the peel surface rough [28,29]. The results of this study showed that, compared with bagging cultivation, the genes involved in the biosynthesis and metabolism of cutin and suberin in the peels of non-bagging-cultivated fruits—CYP94A1 (MD10G1190400), PXG4 (MD13G1029500), and BAT1 (MD08G1058900)—were upregulated. Additionally, the accumulation of suberin component metabolites (5-methyl-tetradecanedioic acid (LMFA01170020), 11-Hydroxy-9-tridecenoic acid (LMFA01050437), and 6-Hydroxypentadecanedioic acid (HMDB0031885)) was also upregulated. This is consistent with the previous research results [30,31]. This result suggests that changes in the expression patterns of genes or metabolites related to the accumulation of peel structural substances may disrupt the orderly development of peel cells, leading to disordered distribution of fruit dots. This is also one of the reasons for the reduced appearance quality of non-bagging-cultivated Fuji apples.

A study on pear fruits reported that the biochemical and transcriptional regulation of the antioxidant system affects pear browning [32]. The results of this study indicated that the dull coloration of the peel under non-bagging cultivation is also affected by the biochemical and transcriptional regulation of the antioxidant system. Peroxidase (MD01G1162700, MD15G1252300) is involved in hydrogen peroxide-related oxidation reactions and can catalyze the oxidation of various substrates such as phenols and amines. The upregulated expression of such genes in the peel catalyzes the oxidation of phenols to quinones, and the quinones further polymerize to form dark substances such as melanin (Table 5). This is because non-bagging-cultivated fruits are more susceptible to mechanical damage and pest infestations, which leads to the upregulated expression of genes related to the synthesis of peel resistance components and the subsequent enhanced expression of genes related to antioxidant reactions.

### 3.3. The Reduction in Appearance Quality Is Related to the Accumulation of Flavonoids in the Fruit Peel

The appearance quality of fruits mainly encompasses two aspects: peel smoothness and peel coloration. Among these, peel coloration is primarily associated with the flavonoid metabolism pathway [33]. Studies have shown that the reduced appearance quality of non-bagging-cultivated fruits is closely linked to differences in the accumulation of flavonoids (Figure 11) [34,35].

Flavonoids are important secondary metabolites in plants and can be subdivided into multiple subclasses, including flavones, flavonols, flavanols, and anthocyanins [36]. Anthocyanins, which belong to a subclass of flavonoids, impart a red color to plants. In contrast, other flavonoid substances—such as flavones, flavonols, and flavanols (excluding anthocyanins)—cause the peel to appear yellow when accumulated in the peel [36,37].

Through transcriptomic and metabolomic analyses (Table 5 and Table 6), this study found that the differential metabolites between non-bagging and bagging cultivation are mainly quercetin and its glycosides (Table 6). These substances belong to the flavonol subclass of flavonoids and are inherently pale yellow or pale green. When accumulated in the peel, they impart colors such as yellow to the peel; meanwhile, flavonols can also interact with other pigments (e.g., anthocyanins) to deepen the peel color [38,39]. This is one of the reasons why non-bagging-cultivated fruits exhibit darker peel coloration and poorer appearance quality. Critically, this upregulation of specific flavonol glycosides identified by metabolomics directly corresponds with and provides a molecular explanation for the significantly higher total flavonoid content measured in the peel of non-bagging-cultivated fruits (Figure 3b). This strong agreement between the physiological biochemistry and metabolomic data solidifies the conclusion that enhanced flavonoid metabolism is a key contributor to the dull coloration phenotype under non-bagging cultivation.

Notably, physiological index determination showed that the contents of chlorophyll and carotenoids in non-bagging-cultivated fruits are higher than those in bagging-cultivated fruits, while the anthocyanin content is lower. However, these key end-product pigments were not identified as differential metabolites in our non-targeted metabolomic profile. This apparent discrepancy can be attributed to the high metabolic flux and dynamic equilibrium within the light-exposed peel. First, the transcriptomic changes indicate accelerated pigment synthesis, which is likely counterbalanced by concurrent degradation processes (e.g., photo-oxidation), preventing significant net accumulation in the metabolomic snapshot. Additionally, the significant alterations we observed in upstream precursors within the flavonoid/phenylpropanoid pathways provide direct metabolic evidence of active pathway modulation. Therefore, the transcriptional signal primarily reflects a change in metabolic dynamics rather than a simple accumulation of final pigments, offering a more nuanced explanation for the pigment ratio imbalance.

### 3.4. Limitations and Future Perspectives

This study provides a comprehensive metabolomic and transcriptomic profile underlying the appearance quality deterioration in non-bagging cultivated apples, identifying key candidate genes and metabolites. The datasets generated herein offer a valuable resource for future studies, which should aim to definitively validate the functions of the proposed candidate genes (e.g., CYP94A1, CML41) and unravel the complex regulatory networks orchestrated by environmental factors, thereby fully elucidating the mechanistic basis of appearance quality in non-bagging cultivated apples.

## 4. Materials and Methods

### 4.1. Plant Materials and Experimental Design

The experiment was conducted in 2022 at the Taidong Base of Shandong Institute of Pomology (36°11′07″ N, 117°06′51″ E), with the experimental site and design being identical to those described in [40]. Briefly, the orchard is characterized by a temperate continental semi-humid monsoon climate and sandy loam soil. The test materials were 13-year-old ‘Tianhong 2’ Fuji apple trees, grafted onto SH dwarfing intermediate rootstock with ‘*Malus robusta*’ as the rootstock base, planted at a spacing of 1.5 m × 3 m (the north–south direction).

A completely randomized design was employed. For the bagging treatment, double-layer paper bags (KM-2, Tokyo, Japan) were applied on 28 May (48 days after full bloom, DAFB) and removed on 8 October (178 DAFB). The non-bagging treatment was left untreated throughout the growing season.

To ensure biological relevance and statistical robustness, three independent biological replicates were used for all analyses. Each biological replicate consisted of a pool of tissues collected from five different trees within the same orchard block. All fruit samples were harvested at commercial maturity on October 28 (198 DAFB).

### 4.2. Sampling Strategy

For each biological replicate (i.e., each pool from five trees), 15 fruits of uniform size, maturity, and without visible blemishes were randomly selected.

For physiological and biochemical analyses (Section 4.3.1, Section 4.3.2, Section 4.3.3, Section 4.3.4 and Section 4.3.5), the peel from 10 fruits was carefully removed using a stainless-steel peeler, immediately mixed to form one composite sample per replicate, flash-frozen in liquid nitrogen, and stored at −80 °C until analysis.

For transcriptomic and metabolomic analyses (Section 4.3.6, Section 4.3.7 and Section 4.3.8), the peel from the remaining 5 fruits per replicate was similarly collected, pooled to create a single composite sample for each replicate, flash-frozen in liquid nitrogen, and stored at −80 °C.

For ultrastructural observation (Section 4.3.2), fresh peel tissues from 3 additional fruits per replicate were collected and processed immediately.

### 4.3. Measurement Methods

#### 4.3.1. Determination of Fruit Appearance Quality

The color of the fruit surface was determined using a CI-410 color difference meter (CI-410, Konica Minolta, Tokyo, Japan). The indexes included L*, a*, b*, C, and h°, where C^2^ = [(a*)^2^ + (b*)^2^]/2, h° = tan^−1^(b*/a*) (a* > 0, b* > 0) or h° = tan^−1^(b*/a*) + 180° (a* < 0, b* > 0) [41].

Fruit Surface Coloring Index = ∑ (number of fruits at all levels × representative grade value)/(total number of fruits × highest value) × 100%. The color grading standard was as follows: Grade 0, 0–5% fruit surface colored; Grade 1, 5–25% fruit surface colored; Grade 2, 25–50% fruit surface colored; Grade 3, 50–75% fruit surface colored; Grade 4, 75–100% fruit surface colored.

Smoothness Index = ∑ (number of fruits at all levels × representative grade value)/(total number of fruits × highest value) × 100%. The Smoothness Index grading standard was as follows: Grade 0, 0–10% smooth fruit surface; Grade 1, 10–30% smooth fruit surface; Grade 2, 30–60% smooth fruit surface; Grade 3, 60–85% smooth fruit surface; Grade 4, 85–100% smooth fruit surface.

Peel samples were randomly collected from the sun-exposed side of the fruits, observed, and photographed in real time under a stereomicroscope (Olympus SZX16, Tokyo, Japan) at 7× magnification. The size of fruit dots was measured, and the length and width of each fruit dot were recorded.

#### 4.3.2. Observation of Peel’s Ultrastructure

Epidermal tissue was cut from the middle part of apple fruits using a single-edge blade to prepare samples with a side length of 5 mm and a thickness of 0.2 mm. The samples were fixed in 4% glutaraldehyde overnight (at 4 °C for over 6 h), then rinsed four times with PBS buffer (pH 6.8, 0.1 M), for 8 min each time. The samples were dehydrated stepwise with 50%, 70%, 80%, and 95% acetone, for 10 min at each concentration; subsequently, they were dehydrated with 100% acetone 3 times, 10 min each time. Finally, embedding, sectioning (70–90 nm), and section retrieval were performed. After staining, the samples were observed with a Hitachi H-7650 transmission electron microscope (TEM).

#### 4.3.3. Determination of Chlorophyll (Chl) and Carotenoid (Car) Contents

Chlorophyll (Chl) and carotenoid (Car) contents were determined according to the method from Zhao et al. (2002) [42] with some modifications. Briefly, 0.5 g of apple peel was extracted in 20 mL of 80% (*v*/*v*) acetone in the dark at 4 °C for 40 h, with occasional shaking during this period. The absorbance (A) of the extract solution was measured at 663, 646, and 470 nm. The content of each pigment was calculated using the following equations, which are based on the specific extinction coefficients established by Wellburn (1994) [43]:

Total Chlorophyll (Chl a + b) (mg/g FW) = (17.32 × A_646_ + 7.18 × A_663_) × V/(1000 × W); (1)

Carotenoid (Car) (mg/g FW) = (1000 × A_470_ − 2.05 × Chl a − 77.6 × Chl b) × V/(1000 × W). (2)

where

Chl a (mg/g FW) = (12.25 × A_663_ − 2.79 × A_646_) × V/(1000 × W)

Chl b (mg/g FW) = (21.50 × A_646_ − 5.10 × A_663_) × V/(1000 × W)

FW refers to fresh weight; V is the volume of the extracting solution (mL); W is the fresh weight of the sample (g).

#### 4.3.4. Determination of Total Anthocyanin Content

Total anthocyanin content was determined as described earlier (Zheng and Tian, 2006) [44]. Briefly, 0.1 g of apple peel was sliced and extracted with 1 mL of HCl-Methanol (95% methanol) (15:85, *v*/*v*) for four hours. The extract was filtered, and the anthocyanin concentration was measured using an ultraviolet spectrophotometer at 530, 620, and 650 nm, respectively (Inacio et al., 2013) [45].

The anthocyanin content was calculated using the following equation:

Anthocyanin Content (nmol/g) = ((A_530_ − A_620_) − 0.1(A_650_ − A_620_))/ε × (V/m) × 10^6^

where C is the anthocyanin content (nmol/g), A is the corrected absorbance at the corresponding wavelength, V is the volume of the extracting solution (mL), m is the fresh weight of the apple peel (g); ε is the extinction coefficient of anthocyanin (4.62 × 10^4^ L·mol^−1^·cm^−1^), and 10^6^ is a conversion factor to nmol/g.

#### 4.3.5. Determination of Total Flavonoid Content

Total flavonoid content was determined following the spectrophotometric method described by Koley et al. and Xue et al. [46,47]. Briefly, a 1 mL aliquot of the extract was pipetted into a 10 mL volumetric flask and mixed with 4 mL of deionized water (dH_2_O), 0.3 mL of 5% sodium nitrite (NaNO_2_) solution, and 0.3 mL of 10% aluminum chloride hexahydrate (AlCl_3_·6H_2_O) solution. The mixture was incubated at room temperature for 5–6 min, after which 2 mL of 1 mol/L sodium hydroxide (NaOH) solution was added. The resulting solution was then diluted to a final volume of 10 mL with dH_2_O, and its absorbance was measured at 510 nm. The results were expressed as rutin equivalent (mg RE/g) [47]. Total flavonoid content was calculated as follows:

Total flavonoid content (mg RE/g FW) = C × V × D/W/1000

where C: total flavonoid concentration in the sample solution, calculated from the rutin standard curve (μg/mL), V: volume of the extracting solution (mL), W: fresh weight of the apple peels (g), D: dilution factor of the sample extract (e.g., 1 if 1 mL of undiluted extract is used for detection), 1000: Conversion factor (convert μg to mg).

#### 4.3.6. The Total RNA Extraction and Transcriptome Sequencing

Total RNA was extracted using the TRIzol reagent (Invitrogen, Carlsbad, CA, USA) according to the manufacturer’s protocol. RNA purity and quantity were evaluated using the NanoDrop 2000 spectrophotometer (Thermo Scientific, Waltham, MA, USA). RNA integrity was assessed using the Agilent 2100 Bioanalyzer (Agilent Technologies, Santa Clara, CA, USA). Then, libraries were constructed using the VAHTS Universal V6 RNA-seq Library Prep Kit according to the manufacturer’s instructions. Transcriptome sequencing and analysis were conducted by OE Biotech Co., Ltd. (Shanghai, China). Paired-end sequencing was performed using an Illumina NovaSeq 6000 (Illumina, San Diego, CA, USA) to obtain sequencing data.

#### 4.3.7. Functional Analysis of DEGs and qRT-PCR Validation

After quality evaluation of the sequencing data, clean data were obtained and aligned to the apple reference genome using HISAT2 2.2.1 software [48,49] to obtain gene expression information of the samples. Differential expression analysis was conducted using DESeq2 1.48.2 software, and gene functional annotation was conducted using GO and KEGG databases to identify the metabolic pathways involved in DEGs.

Six significantly DEGs with |log_2_ Fold Change| ≥ 1 were randomly selected. Total RNA was extracted using the Plant RNA Kit (OMEGA bio-tek, Norcross, GA, USA), and reverse transcription and qRT-PCR were performed with the TransScript One-Step gDNA Removal and cDNA Synthesis SuperMix (TransGen Biotech Co., Ltd., Beijing, China). The primer sequences for qRT-PCR are listed in Table S3. The qRT-PCR technique was used to verify the expression changes in the selected DEGs [50].

#### 4.3.8. Extraction, Determination, and Analysis of Metabolites

Peel samples were ground in liquid nitrogen and then placed into an extraction solution for extraction. The metabolic samples were analyzed using a UPLC-MS system (ultra-performance liquid chromatography-tandem mass spectrometry system) (UPLC-MS), and metabolites were qualitatively identified based on secondary mass spectrometry information.

The obtained mass spectrometry data were subjected to principal component analysis (PCA) and sample correlation analysis to evaluate the reproducibility and degree of difference among samples. Orthogonal partial least squares discriminant analysis (OPLS-DA) was used to analyze metabolites and screen for significantly different metabolites. The KEGG database and HMDB (Human Metabolome Database) were employed for functional analysis of the differential metabolites, to determine the specific biological pathways involved in these metabolites.

### 4.4. Statistical Analysis

All experiments were conducted using the three independent biological replicates as described in Section 4.2 The presented values represent the mean ± standard error (S.E.) of these three replicates. The test results were plotted with Sigma Plot 10.0 (Systat Software, Inc., San Jose, CA, USA), and statistical analysis was conducted using Data Processing System software 15.10 (DPS; Hangzhou, Zhejiang, China). Significant differences between treatments were determined using Duncan’s multiple range test at *p* < 0.05.

## 5. Conclusions

Compared with bagging cultivation, non-bagging cultivation of Fuji apples exhibits rougher peels, duller coloration, and significantly reduced fruit appearance quality. This decline is attributed to three major factors: first, the peels of non-bagging-cultivated apples possess higher chlorophyll and carotenoid contents but lower anthocyanin levels, disrupting the normal pigment balance and impairing color development; secondly, key genes involved in cutin, suberin, and wax biosynthesis—such as CYP94A1 (MD10G1190400), PXG4 (MD13G1029500), and BAT1 (MD08G1058900)—are significantly upregulated, accompanied by increased accumulation of suberin metabolites including 5-methyltetradecanedioic acid (LMFA01170020), 11-hydroxy-9-tridecenoic acid (LMFA01050437), and 6-hydroxypentadecanedioic acid (HMDB0031885), collectively leading to peel roughness; thirdly, flavonoid and flavonol metabolites—such as quercetin (HMDB0005794) and rutin (HMDB03249)—accumulate to higher levels, further contributing to the dullness of the fruit peel. Together, these findings enhance our understanding of the molecular mechanisms governing fruit quality under non-bagging cultivation and provide a theoretical basis for improving apple production practices without bagging.

## Figures and Tables

**Table 1:** Effects of non-bagging and bagging cultivation on fruit dot characteristics in Fuji apple.

Treatment	Fruit Dot Characteristics		
	Fruit Dot Density (No./cm^2^)	Length (μm)	Width (μm)
non-bagging	4.71 ± 0.37 b	881.95 ± 2.75 b	696.04 ± 1.72 a
bagging	5.15 ± 0.19 a	640.14 ± 3.26 a	446.17 ± 0.70 a

Note: Data are presented as the mean ± standard error (SE) (n = 3). Different lowercase letters within the same column indicate significant differences between treatments as determined by Duncan’s test (*p* < 0.05).

**Table 2:** Effects of non-bagging and bagging cultivation on peel coloration and smoothness indices, and chromaticity parameters in Fuji apple.

Treatment	Coloring Index (%)	Finish Index (%)	Chromatic Aberration				
			L*	a*	b*	C	h°
non-bagging	79.55 ± 3.21 b	70.45 ± 2.89 b	4.41 ± 3.71 b	21.70 ± 2.75 b	14.12 ± 1.72 a	18.36 ± 1.69 b	1.33 ± 0.33 b
bagging	90.91 ± 2.15 a	86.36 ± 2.54 a	5.15 ± 1.93 a	30.49 ± 3.26 a	13.40 ± 0.70 a	23.57 ± 2.03 a	2.14 ± 0.35 a

Note: Data are presented as the mean ± SE (n = 3). Different lowercase letters within the same column indicate significant differences between treatments as determined by Duncan’s test (*p* < 0.05). L* represents brightness, with larger values indicating greater brightness and better peel smoothness; a* and b* are chromaticity indicators. A higher positive a* value indicates a deeper red color, while a higher negative value indicates a deeper green color; A higher positive b* value indicates a deeper yellow color, while a higher negative value indicates a deeper blue color. C represents the chroma value, with higher values indicating stronger coloration; h° represents the comprehensive chroma value (higher values indicate higher comprehensive chroma).

**Table 3:** Effect of non-bagging and bagging cultivation on chlorophyll pigment content in Fuji apple peel.

Treatment	Chloroplast Pigment (mg/g FW)	
	Chl	Car
non-bagging	0.019 ± 0.001 a	0.016 ± 0.001 a
bagging	0.002 ± 0.001 b	0.008 ± 0.000 b

Note: Data are presented as the mean ± SE (n = 3). Different lowercase letters within the same column indicate significant differences between treatments as determined by Duncan’s test (*p* < 0.05). FW: Fresh weight.

**Table 4:** Summary of sequencing data quality for non-bagging and bagging-cultivated Fuji apple peels.

Sample	Non-Bagging-1	Non-Bagging-2	Non-Bagging-3	Bagging-1	Bagging-2	Bagging-3
Raw Reads (G)	7.39	7.76	7.57	6.80	7.50	7.22
Clean reads (G)	6.53	6.92	6.77	6.08	6.68	6.56
Q30 (%)	95.00	94.17	94.72	94.50	93.80	94.39
GC content (%)	49.22	48.63	48.68	48.60	48.73	48.54

**Table 5:** Statistics of candidate genes related to peel appearance quality deterioration.

Class	Gene ID	Gene Symbol	Description	Log_2_ Fold Change	*p*-Adjust	Regulate
secondary metabolites	MD14G1210700	CYP75B2	Flavonoid 3′-hydroxylase	1.20	7.13 × 10^−88^	Up
	MD13G1046900	LAR	Putative leucoanthocyanidin reductase	1.45	3.67 × 10^−4^	Up
	MD01G1162700	PER52	peroxidase P7-like	2.40	5.14 × 10^−46^	Up
	MD15G1252300	PER72	peroxidase 72	1.79	7.46 × 10^−8^	Up
	MD03G1013200	PER53	peroxidase A2-like	5.66	2.77 × 10^−4^	Up
	MD05G1320800	PPO	polyphenol oxidase, chloroplastic-like	−1.61	4.38 × 10^−2^	Down
	MD14G1172500	cao1	amine oxidase 4	−4.13	2.79 × 10^−2^	Down
	MD02G1136000	CYP84A1	cytochrome P450 84A1-like	−5.64	7.48 × 10^−13^	Down
	MD07G1300500	COMT1	caffeic acid 3-O-methyltransferase	1.57	1.86 × 10^−4^	Up
	MD13G1090300	CCD4	carotenoid cleavage dioxygenase 4	−1.44	3.25 × 10^−7^	Down
	MD10G1194200	NCED1	9-cis-epoxycarotenoid dioxygenase NCED3, chloroplastic-like	2.91	1.23 × 10^−3^	Up
Lipids	MD11G1023100	LOX2.1	linoleate 13S-lipoxygenase 2-1, chloroplastic-like	1.62	3.98 × 10^−108^	Up
	MD16G1113200	LOX6	lipoxygenase	1.15	4.93 × 10^−2^	Up
	MD10G1190400	CYP94A1	cytochrome P450 94A1-like isoform X2	3.11	1.17 × 10^−26^	Up
	MD08G1058900	BAT1	brassinosteroid-related acyltransferase 1-like	3.30	6.62 × 10^−5^	Up
	MD13G1029500	PXG4	probable peroxygenase 4 [Malus domestica]	2.51	2.97 × 10^−6^	Up
Carbohydrate	MD16G1042700	RBCS	ribulose bisphosphate carboxylase small chain, chloroplastic-like	−1.79	2.17 × 10^−268^	Down
	MD11G1295000	rbcL	ribulose-1,5-bisphosphate carboxylase/oxygenase large subunit (chloroplast)	−1.18	2.52 × 10^−17^	Down
	MD13G1093700	GOLS1	galactinol synthase 1-like	−2.12	4.51 × 10^−142^	Down
	MD13G1186100	ADG2	glucose-1-phosphate adenylyltransferase large subunit 3	4.19	4.23 × 10^−127^	Up
	MD04G1056200	BAM3	beta-amylase 3	2.81	1.85 × 10^−14^	Up
	MD14G1167100	At4g24780	probable pectate lyase 18	3.28	1.76 × 10^−108^	Up
	MD02G1067000	PME53	probable pectinesterase 53	4.42	2.39 × 10^−2^	Up
	MD14G1128000	At1g64390	endoglucanase 6	1.21	7.52 × 10^−22^	Up
	MD05G1006400	SPS4	probable sucrose-phosphate synthase 4	1.11	2.09 × 10^−2^	Up
Environmental adaptation	MD07G1292500	CNGC1	cyclic nucleotide-gated ion channel 1-like	5.38	5.47 × 10^−2^	Up
	MD08G1037100	CML41	probable calcium-binding protein CML41 isoform X1	4.89	1.22 × 10^−2^	Up
	MD02G1271900	EIX2	LRR receptor-like serine/threonine-protein kinase GSO2	3.98	2.00 × 10^−2^	Up
	MD17G1257900	CML27	probable calcium-binding protein CML23	3.11	1.53 × 10^−6^	Up
Energy metabolism 00710	MD15G1091000	ME1	NADP-dependent malic enzyme isoform X2	1.38	4.31 × 10^−54^	Up
	MD02G1011600	AHA8	ATPase 8, plasma membrane-type	4.59	1.54 × 10^−2^	Up
	MD13G1247200	PMA4	plasma membrane ATPase 4-like	1.12	2.88 × 10^−23^	Up
Membrane transport-ABC	MD17G1042800	ABCB15	ABC transporter B family member 15-like	4.98	9.74 × 10^−38^	Up
	MD10G1268400	ABCB9	ABC transporter B family member 9-like	1.00	5.39 × 10^−4^	Up

**Table 6:** Statistics of DEMs related to peel darkening.

Class	Compound ID	Metabolite	VIP	FC	Type
Lipids and lipid-like molecules	HMDB0006928	δ8,14-Sterol	1.636	1.627	Up
	HMDB0006591	Lactosamine	2.724	4.278	Up
	HMDB0029315	Asparagoside B	2.199	2.055	Up
	LMST01010173	cholesteryl α-D-glucoside	3.816	2.626	Up
	HMDB0006867	S-(3-Methylbutanoyl)-dihydrolipoamide-E	4.016	6.058	Up
	LMPK12112185	Quercetin 3-apiosyl-(1->2)-α-L-arabinopyranoside	1.022	3.398	Up
	LMPK12112087	Quercetin 3-apiosyl-(1->2)-glucoside	2.578	2.759	Up
	LMPK12112155	Quercetin 3-(2‴-p-coumarylsambubioside)-7-glucoside	1.645	2.569	Up
	LMFA01170020	5-methyl-tetradecanedioic acid	1.635	6.452	Up
	LMFA01050437	11-Hydroxy-9-tridecenoic acid	1.227	6.255	Up
	HMDB0031885	6-Hydroxypentadecanedioic acid	2.440	5.986	Up
	HMDB0006294	16-Hydroxyhexadecanoic acid	2.242	0.730	Down
Organic acids and derivatives	HMDB00191	L-Aspartic acid	3.460	0.492	Down
	HMDB0000695	Ketoleucine	1.473	0.394	Down
	HMDB0003705	Phosphoguanidinoacetate	1.014	0.304	Down
	HMDB0012265	N-Carbamoylsarcosine	3.245	0.175	Down
	HMDB0001325	N6,N6,N6-Trimethyl-L-lysine	1.43	0.170	Down
	HMDB0000026	Ureidopropionic acid	3.919	0.320	Down
	HMDB0000168	L-Asparagine	11.491	0.204	Down
	HMDB0006483	D-Aspartic acid	6.139	0.350	Down
	HMDB0000193	Isocitric acid	2.918	0.276	Down
	HMDB00744	Malic acid	13.479	0.723	Down
	HMDB0000094	Citric acid	4.323	0.322	Down
Organic oxygen compounds	HMDB0000143	D-Galactose	6.455	0.497	Down
Phenylpropanoid and polyketide	HMDB03249	Rutin	11.034	2.807	Up
	LMPK12020046	Catechin 7-O-β-D-xyloside	1.020	3.886	Up
	HMDB0255461	Naringin dihydrochalcone	1.212	0.014	Down
	HMDB0000567	Cinnamic acid	2.489	0.372	Down
	HMDB0038808	Luteolin 3′-(3″-acetylglucuronide)	1.620	3.039	Up
	HMDB0037948	Catechin 5-glucoside	1.526	0.207	Down
	HMDB0005794	Quercetin	4.680	1.494	Up
	HMDB37368	Quercetin 3-O-malonylglucoside	2.244	1.994	Up
	LMPK12112097	Quercetin 3-neohesperidoside	11.497	3.119	Up
	HMDB32616	Sinapic acid	1.175	0.426	Down
Organoheterocyclic compounds	HMDB0001264	Dehydroascorbic acid	1.639	0.303	Down
Nucleoside	HMDB0001202	dCMP	1.647	0.320	Down
	HMDB0001044	2′-Deoxyguanosine 5′-monophosphate	1.018	0.358	Down
	HMDB00302	UDP-D-galactose	1.001	1.735	Up

## Data Availability

Data is contained within the article or Supplementary Materials.

## Supplementary Materials

The following supporting information can be downloaded at: https://www.mdpi.com/article/10.3390/plants14213339/s1, Table S1: Identification of total metabolites in nonbagging and bagging apple peels; Table S2: Significantly different metabolites in nonbagging vs. bagging apple peels; Table S3: qRT-PCR primer sequence.
